# Glabridin Attenuates the Retinal Degeneration Induced by Sodium Iodate *In Vitro* and *In Vivo*

**DOI:** 10.3389/fphar.2020.566699

**Published:** 2020-10-15

**Authors:** Kaung Htet Aung, Hua Liu, Zongwen Ke, Shuang Jiang, Jianhua Huang

**Affiliations:** ^1^Department of Ophthalmology, Third Affiliated Hospital of Jinzhou Medical University, Jinzhou, China; ^2^Graduate School of Jinzhou Medical University, Jinzhou, China; ^3^Key Laboratory of Surgery of Liaoning Province, First Affiliated Hospital of Jinzhou Medical University, Jinzhou, China

**Keywords:** age-related macular degeneration, apoptosis, glabridin, oxidative stress, retinal pigment epithelial (RPE) cells, sodium iodate

## Abstract

**Background:**

Age-related macular degeneration (AMD) is one of the major causes of blindness in the elderly over the age of 60. AMD is divided into dry AMD and wet AMD. Although there are certain treatment methods for wet age-related macular degeneration (AMD), there are no effective treatments for dry AMD yet, and finding new drugs or treatment methods for dry AMD has become a priority. For this purpose, this study explored Glabridin (Glab), an isoflavane found in the root extract of licorice, which has never been investigated in relation to eye diseases.

**Purpose:**

To investigate the effect of Glab on the sodium iodate (NaIO_3_) induced retinal degeneration *in vitro* and *in vivo*.

**Methods:**

*In vitro*, cell viability and cytotoxicity were tested with methylthiazolyldiphenyl‐tetrazolium bromide (MTT) assay among the groups of ARPE-19 cells. The cell apoptosis was tested with Hoechst 33342 staining and flow cytometry. The level of Reactive oxygen species (ROS) was measured to check the effect on oxidative stress. The protein expressions of phosphorylation of ERK1/2 and p38 were detected by Western blotting. *In vivo*, C57BL/6J mice were pretreated with Glab intraperitoneally for one week and continued for 4 weeks. NaIO_3_ was given to mice through tail vein intravenous injection after 1 week of Glab administration. The retinas of mice were monitored by Optical coherence tomography (OCT) and electroretinography (ERG) at 1w, 2w, 3w, and 4w, respectively, followed by H&E staining.

**Results:**

*In vitro*, the Glab protected the retinal pigment epithelial (RPE) cells against oxidative stress and apoptosis by inhibiting phosphorylation of ERK1/2 and the p38 MAPK pathway. *In vivo*, Glab significantly prevented retinal damage by stopping the progression of retinal degeneration and reducing the formation of deposits on the RPE layer induced by NaIO_3_. According to the findings of electroretinogram (ERG), Glab helped to maintain the normal function of the retina.

**Conclusion:**

Glabridin has a protective effect against retinal degeneration. It is suggested that Glab be further investigated for the treatment of retinal degeneration diseases.

## Introduction

Among many retinal diseases, age-related macular degeneration (AMD), is a threat to the vision of the increasing elderly population, due to the devastating characteristics of irreversible visual damage, even blindness, incomplete knowledge of cause or mechanism, and a lack of effective treatment. AMD is generally defined as the loss of central vision, characterized by the progressive degeneration of the macula (Lim et al., 2012; Handa et al., 2017). There are two types of AMD: wet AMD, which can cause the rapid central vision loss because of abnormal blood vessels growing into the macula and scarring it by leaking blood or fluid; and dry AMD, which is the slow loss of vision as the gradual deterioration of macula is caused by an accumulation of small white or yellowish deposits, called drusen. Dry AMD affects almost 90% of AMD cases and it is even considered that the dry form could be a risk factor or precursor state for wet AMD because most AMD cases typically start in dry form and progress to wet form (Nowak, 2006; Li et al., 2018). According to an European report from 2017, although the prevalence of early AMD increases with age, the prevalence of late AMD decreases, probably because of healthier lifestyles and the treatment of wet AMD with anti-vascular endothelial growth factor (VEGF). Although the implementation of anti-VEGF treatment seems to be decreasing the prevalence of AMD, it is predicted that the number of affected persons will still increase in the next two decades (Colijn et al., 2017). To further decrease the prevalence of AMD, discovering the treatment options for dry AMD seems to be the appropriate solution since it remains untreatable.

According to previous studies, dry AMD is mainly characterized by the formation of drusen that occurs due to toxic accumulations, either within the retinal pigment epithelial (RPE) cell or at the RPE-BrM (Bruch’s membrane) interface. Along with other risk factors (such as aging, smoking, and genetic factors, etc.), oxidative stress and other stresses cause RPE and photoreceptor (PR) cell death, leading to toxic accumulations and resulting in sub-RPE drusen deposits (Ambati and Fowler, 2012; Bowes Rickman et al., 2013). A major objective in the treatment of AMD is to prevent the formation of drusen on the retina as soon as the disease is diagnosed, avoiding the oxidative stress and apoptosis of RPE cells, since the removal of drusen after formation is not yet possible.

Flavonoids have been reported to have a variety of biological activities, such as anti-cancer, anti-inflammatory, and cardiovascular protective effects. Some researchers have studied treatment with certain flavonoids such as anthocyanins and xanthophylls, observing that they can significantly reduce the level of ROS, the phosphorylation levels of ERK1/2 and p38 in ARPE-19 cells, and increase cell viability (Silván et al., 2016). Other reports have proven that flavonoids can protect human retinal pigment epithelial cells and retinal ganglion cells from oxidative-stress induced death (Maher and Hanneken, 2005; Hanneken et al., 2006). Many types of polyphenols and flavonoids are considered to have preventive and therapeutic effects in age-related eye diseases (Bungau et al., 2019).

Glabridin (Glab) is a bio-available isoflavan, a type of isoflavonoid, extracted from licorice (*Glycyrrhiza glabra* L.) root. Many previous studies have verified that Glab possesses strong antioxidant activity, anti-inflammation activity, and neuroprotective properties. Recent studies have also indicated that Glab can prevent injuries in major organs such as cardiotoxicity, endothelial dysfunction, and acute lung-injury (Carmeli and Fogelman, 2009; Zhang et al., 2017; Wang et al., 2018; Huang et al., 2019). The effect of Glab were also investigated in several cancer cells and Glab was considered to possess the ability to regulate cell survival and cell proliferation (Huang et al., 2014; Zhang et al., 2018). However, there are to date, no reports about the effects of Glabridin on any retinal diseases.

The models of sodium iodate (NaIO_3_)-induced retinal degeneration were extensively investigated in different concentrations, duration, and species in the past. In mouse models, systemic delivery has been used as an effective method to induce retinal degeneration associated with the regional loss of retinal pigment epithelium (RPE), imitating most of the features of dry AMD (Mizota and Adachi-Usami, 1997; Enzmann et al., 2006; He et al., 2019). A concentration range of 20~30mg/kg of NaIO_3_ seems to be a suitable dosage in inducing central retinal degeneration, with the characteristics of RPE death and outer nuclear layer (ONL) apoptosis peaking at day 3 and continuing to decline until day 28 (Zeiss, 2013; Wang et al., 2014). *In vitro* studies for NaIO_3_-induced retinal pigment epithelial cell dysfunction indicate that the concentration range of 1000~2000 μg/ml could induce oxidative stress and apoptosis in RPE cells without causing massive cell death as in high dose exposure (Zhang et al., 2016). In this study, the concentration of 25mg/kg of NaIO_3_ was given to the C57BL/6J mice through tail vein intravenous injections and the concentration of 1200 μg/ml of NaIO_3_ was used to induce RPE cell dysfunction to create the appropriate model of dry AMD. The effect of Glabridin on these models was then investigated *in vitro* and *in vivo*.

## Materials and Methods

### Cells and Reagents

Human retinal pigment epithelial cells (ARPE‐19) were purchased from American Type Culture Collection (ATCC, Manassas, VA, USA). The Glabridin (purity ≥ 98%) was purchased from the National Institute for Food and Drug Control (Nanjing, China). Sodium iodate (NaIO_3_) was purchased from Sigma Industrial Company. 3-(4,5-dimethylthiazol-2-yl)−2,5-diphenyl tetrazolium bromide (MTT), Hoechst 33342, and ROS DCFH-DA staining were purchased from Beyotime. Trypsin-EDTA, DME/F-12, penicillin-streptomycin (PS), and Fetal Bovine Serum (FBS) were purchased from Gibco and Hyclone (Logan, Utah). DMSO was purchased from Sigma-Aldrich. The materials required for western blotting were purchased from Bio-Rad and Biosharp. The transfer Membranes were purchased from Immobilon-P. Anti-ERK1/2, anti-p-ERK1/2, anti-p38, anti-p-p38, anti-GAPDH, and anti-β-tubulin were purchased from Abcam. All the essential materials and machines were provided by the Laboratory of the Ophthalmology Department of Jinzhou Medical University.

### Cell Culture

Human retinal pigment epithelial cells (ARPE-19) passages 5-10 were used for all experiments. Cells were cultured in 10 cm^2^ cell culture dishes in DME-F12 with 10% of FBS and 2% of penicillin-streptomycin, and incubated at 37 °C with 5% CO_2_ humidified atmosphere. The medium was renewed every 2 days.

### Cell viability assay

The colorimetric 3-(4,5-dimethylthiazol-2-yl)-2,5-diphenyl tetrazolium bromide (MTT) assay was used to check cell viability. ARPE-19 cells were cultured into 96 wells plate and divided into the control group, sodium iodate (NaIO_3_) group, treatment group (Glab+ NaIO_3_), and Glabridin (Glab) only group. In the control group, cells were treated only with DME-F12. NaIO_3_(1200 μg/ml) was given to the NaIO_3_ group and treatment group. At the same time, different concentrations of Glabridin (2, 4, 8, 16 μM) were given to the treatment group and Glab only group. After 24 h of incubation, the absorbance cell viability was evaluated by spectrophotometrically using a microplate reader (Bio-Rad, iMark™) at 490 nm.

### Hoechst 33342 Staining

ARPE-19 cells were seeded into 6-wells plate. The cells were treated with NaIO_3_(1200 μg/ml) and Glab(8 μM) and incubated for 24 hrs. After 24hrs, the wells were washed with PBS three times and then stained with 2ml of 1μg/ml Hoechst 33342 and incubated for 30 mins. This whole procedure was performed in the dark to prevent photoquenching of the fluorescence reagent. The cells were washed with PBS three times after incubation. A LAS 4.4 Imaging System was used to take the images. ImageJ software was used to quantify the condensed nuclei in each group. Each group was normalized to the control group (control was taken as 2%, which is the same percentage as the control group in the flow cytometry results, which enabled easy comparison).

### Flow Cytometry

FITC Annexin V Apoptosis Detection Kit with 7-AAD was purchased from BioLegend. In order to test the antiapoptotic effect of Glabridin on ARPE-19 cells treated with Sodium iodate (NaIO_3_), ARPE-19 cells were seeded into 6-well plates, treated with NaIO_3_(1200 μg/ml) and Glab(8μM) and incubated for 24 hrs. Then, the cells were washed with PBS twice, transferred to 1.5 ml microcentrifuge tubes, centrifuged at 4500rpm for 5 mins, and then resuspended in 100 μl of Annexin V Binding Buffer. The FITC Annexin V(5 μl) and 7-AAD Viability Staining Solution (5 μl) were then added to each tube. The cells were gently vortexed and incubated for 15 mins at room temperature in the dark. We then added 400 μl of Annexin V Binding Buffer to each tube. The apoptosis of the cells was analyzed by flow cytometry with NovoExpress Software with proper machine settings.

### Measurement of ROS production

Reactive Oxygen Species (ROS) production was assessed using Dichloro‐dihydro‐fluorescein diacetate (DCFH‐DA) Reagent. ARPE-19 cells were treated with NaIO_3_(1200 μg/ml) and Glab(8 μM) for 24hrs. The staining procedure was performed in the dark according to the instructions of the DCFH-DA Cellular ROS assay kit. The cells were washed with PBS 3 times before and after incubation with DCFH-DA. The fluorescence images were obtained by using the LAS 4.4 Imaging System. The fluorescence intensity of ROS was measured by using ImageJ software and normalized to the control (control was taken as 100%). The raw values of each group were divided by the mean value of the control groups and multiplied by 100.

### Western Blot Analysis

ARPE-19 cells divided into control (DME-F12), NaIO_3_ (1200μg/ml), and NaIO_3_+Glab (2, 4, 8μM) and washed with ice-cold PBS, lysed with RIPA buffer and PMSF, placed in 1.5ml microcentrifuge tubes, vortexed for 20 secs, and then cells were sonicated with 20kHz for 10 secs three times. The tubes were kept on ice throughout the whole procedure. After sonication, cell lysate mixtures were centrifuged at 4°C at 12000 rpm for 20 mins. Then, the supernatant protein lysate was transferred to a fresh tube on ice. Equal concentration of protein was determined with a BCA protein assay kit according to the manufacturer’s instructions, and the samples were denatured by boiling in loading buffer and PBS at 100°C for 5 mins. 10μl of each sample was loaded onto an SDS-PAGE gel, run, and then electrotransferred to the PVDF membrane. The phosphorylation of ERK1/2 and P38 were determined by Western blotting with their respective phospho-specific antibodies while GAPDH and β-tubulin were used as total protein control. The intensities of the bands were measured by using ImageJ software.

### Animals and Drug Administration

30 Male C57BL/6J mice were purchased from Beijing Vital River Laboratory Animal Technology Co. Ltd., Beijing, China. The mice had free access to water and standard diet and were maintained in 22–24°C with a 12 hr light-dark cycle. Mice were randomly allocated and 5 mice were housed per group. All animals were randomly grouped, resulting in an equal number of sample sizes. The group size in our experiments was chosen based upon our previous experience or studies using similar experimental protocols. All the studies were designed to generate groups of equal size, using randomization and blinded analysis. After 2 weeks of environmental adaptation, mice were injected intraperitoneally with Glabridin (Ip, 20mg/kg.d) daily for 1 week in the treatment group before NaIO_3_ injection. After 1week, NaIO_3_(25mg/kg) was injected into the tail veins of both the NaIO_3_ group and the treatment group. We continued to administer Glabridin (Ip, 20mg/kg.d) to the treatment group daily for 4 weeks. Every effort was made to avoid animal suffering and minimize the number of animals used. All animal experiments were reviewed and approved by the Experimental Animal Ethics Committee of Jinzhou Medical University and conformed to the Guide for the Care and Use of Laboratory Animals published by the USA National Institutes of Health (Publication, 8th Edition, 2011).

### Optical Coherence Tomograph (OCT) Imaging

Mice were anesthetized by intraperitoneal injection of phenobarbital (0.9mg/kg), and their pupils were dilated with topical 2.5% phenylephrine. To protect the corneas during and after the procedure, an ofloxacin eye drop was used. OCT images were recorded on days 7, 14, 21, and 28 after the intravenous Sodium iodate Injection using the Micron IV retinal imaging camera system (Phoenix Research Laboratories, Pleasanton, CA). The thickness of the retina from OCT images was measured using ImageJ software.

### Electroretinography (ERG)

Electroretinograms (ERGs) were recorded on days 7, 14, 21, and 28 after the Sodium iodate (NaIO_3_) injection. Mice were maintained in a completely dark room for 6 hours and then anesthetized with phenobarbital (0.9mg/kg), and their pupils were dilated with topical 2.5% phenylephrine, and we used an ofloxacin eye drop (as described above). Clean ERGs recording procedures were performed following the instructions of Phoenix Research Labs by using a Micron IV-1 machine from Phoenix Research Labs. Different flash strengths were used as a stimulus that enabled us to observe changes in the function of retinas and appropriate flash strengths (4.4, 5.6, 6.2 cds/m^2^) were used for further observations. The a-wave amplitude was measured from the baseline to the trough of the a-wave. The b-wave amplitude is generally measured from the trough of the a-wave to the peak of the b-wave.

### Histological Analysis (H&E Staining)

After monitoring for 28 days, the mice were euthanized for histological analyses. The eyes of the mice were enucleated, embedded in paraffin, and retinal cross section tissues of 5 μm were sliced for Haematoxylin and eosin (H&E) staining. Images of the stained tissues were taken under a microscope. The thickness of the retinal layers from H&E images was measured using ImageJ software.

### Data and Statistical Analysis

All data were included and no outliers were excluded in data analysis and presentation.

The group size in this study represents the number of independent values, and the statistical analysis was undertaken using independent values. We examined cell viability, Relative ROS, Retinal thickness in OCT results, and ONL thickness in the H&E results. Western blotting density analyses were normalized to the control group for comparison and quantification (control group was taken as 100%). This data transformation was done by dividing each raw value by the value of the mean of the control values, which was then converted to a percentage. All statistical analyses were calculated with One-Way ANOVA (for comparison among three or more groups), followed by LSD and Dunnett’s T3 *post hoc* tests with the P value < 0.05 considered as the threshold for statistical significance (with F achieved P < 0.05 and no significant variance inhomogeneity), using IBM SPSS Statistics 23 for Mac OS software.

## Results

### Glabridin Reduced Sodium Iodate Induced ARPE-19 Cell Death *In Vitro*

**Figure 1:**
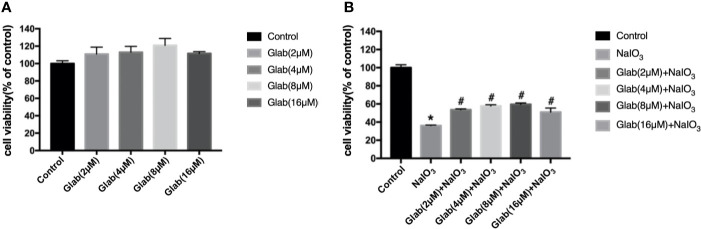
Effects of Glabridin on the viability of ARPE‐19 cells *in vitro*. **(A)** methylthiazolyldiphenyl‐tetrazolium bromide (MTT) assay showed that Glabridin had no toxic effects on ARPE‐19 cells at concentrations of 2, 4, 8 μM. **(B)** MTT assay showed that Sodium iodate(NaIO_3_) group, cells treated with NaIO_3_(1200μg/ml), significantly decreased the viability of ARPE-19 cells compared with the control group (*P < 0.05) and Glabridin at concentrations of 2, 4, 8 μM significantly increased the viability of ARPE‐19 cells treated with sodium iodate, compared with NaIO_3_ group (^#^*P < *0.05).

A 3-(4,5-dimethylthiazol-2-yl)-2,5-diphenyl tetrazolium bromide (MTT) assay was used to evaluate the cytotoxicity of Glabridin. ARPE-19 cells were treated with the different concentrations of Glab (2, 4, 8, 16 μM) in 96 wells plate and incubated for 24 h. The results showed that the concentrations of Glab (2, 4, and 8μM) did not show toxicity in ARPE-19 cells (**Figure 1A**). To test the effects of Glab on NaIO_3_-induced cell death, ARPE-19 cells were treated with Glab (2, 4, 8, 16μM) and NaIO_3_(1200μg/ml) at the same time in 96 wells plate and were incubated for 24h. The results showed that treatment of 1200μg/ml Sodium iodate (NaIO_3_) significantly reduced the cell viability compared with the control group, while treatment with 2, 4, and 8μM Glab significantly attenuated NaIO_3_-induced cell death compared with the NaIO_3_ group (**Figure 1B**). In the concentration of Glab (16μM), the cell viability slightly decreased compared with Glab (8μM), which may mean that the toxicity of the drug appeared in the higher concentrations, and therefore, this concentration(16μM) was not selected for the next experiments.

### Glabridin Attenuated Sodium Iodate Induced Apoptosis in ARPE-19 Cells

**Figure 2:**
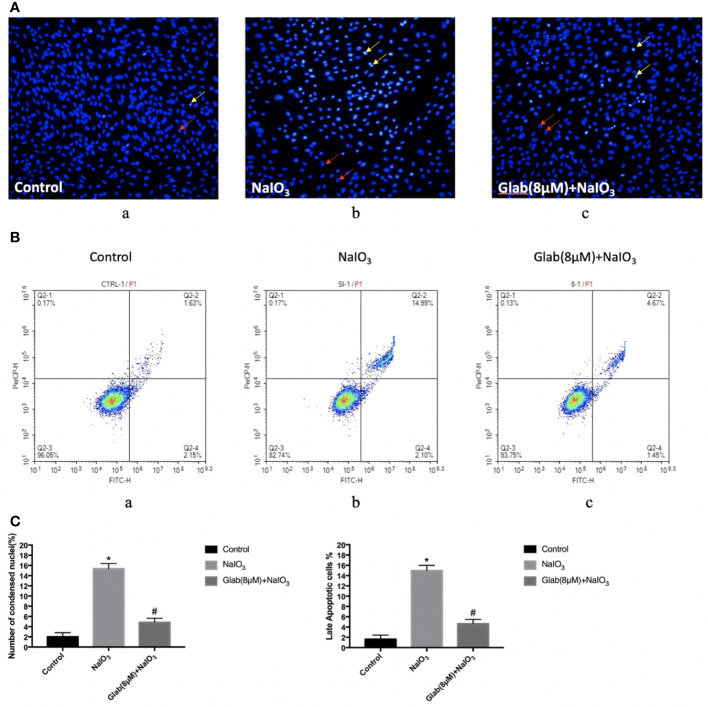
Effects of Glabridin on apoptosis of ARPE‐19 cells *in vitro*. **(A)** Hochest 33342 staining of ARPE‐19 cells treated with sodium iodate for 24 h. Yellow arrows indicate condensed core vs. Red arrows indicate normal core. a, retinal pigment epithelium (RPE) treated with DME-F12 (Control); b, RPE treated with sodium iodate(1200μg/ml) (NaIO_3)_; c, RPE treated with Glabridin(8μM) plus NaIO_3_ (Glab(8μM)+ NaIO_3_). **(B)** Flow cytometry analysis of apoptosis of ARPE‐19 cells treated with NaIO_3_ at a concentration of 1200 μg/ml. The percentage of cells in each quadrant is presented. a, retinal pigment epithelium (RPE) treated with DME-F12 (Control); b, RPE treated with sodium iodate (NaIO_3_); c, RPE treated with Glabridin(8μM) **(C)** Statistical analysis in Hoechst33342 and flow cytometry results; a, Sodium iodate significantly increased the number of condensed nuclei compared with control (*P < 0.05), while Glab treatment significantly decreased the number of condensed nuclei compared with NaIO_3_ group (^#^*P < *0.05). b, Q2-2 quadrant (late apoptosis) of **Figure 2B** is compared. Sodium iodate significantly increased late apoptosis of ARPE‐19 cells compared with the control (*P < 0.05), while Glabridin treatment significantly decreased the late apoptosis of ARPE‐19 cells treated with NaIO_3_, compared with the NaIO_3_ group (^#^*P < *0.05).

In order to test the anti-apoptotic effect of Glab, ARPE-19 cells were treated with NaIO_3_(1200μg/ml) and Glab(8μM) at the same time, and incubated for 24 h, and stained with Hoechst 33342 subsequently. NaIO_3_ group has a significantly greater number of apoptotic cells with condensed nuclei (15.37%), compared with the control group (2%). However, the number of apoptotic cells in the treatment group with Glab (8μM) were significantly fewer to 4.84%, compared with the NaIO_3_ group.(**Figures 2A, Ca**). To confirm the anti-apoptotic effect of Glab in NaIO_3_-induced ARPE-19 cells, cells were treated with NaIO_3_ (1200 μg/ml) and Glab (8μM), incubated for 24h. We used FITC Annexin V Apoptosis Detection Kit with 7-AAD staining to measure the percentages of apoptotic cells with flow cytometry. The percentage of late apoptotic cells in the control group was 1.63% and increased to 14.99% in the SI group (Q2-2 quadrants in **Figures 2Ba, b**). In the treatment group with Glab (8μM), the percentage of late apoptotic cells was reduced to 4.67% (Q2-2 quadrant in **Figure 2Bc**). This flow cytometry result showed that treatment with Glabridin could reduce late apoptotic cells in ARPE-19 cells induced by NaIO_3_. Statistical analysis of both Hoechst33342 and flow cytometry proved that Glab has the anti-apoptotic effect on NaIO_3_-induced ARPE-19 cells (**Figure 2C**).

### Glabridin Attenuated Sodium Iodate Induced ROS Production

**Figure 3:**
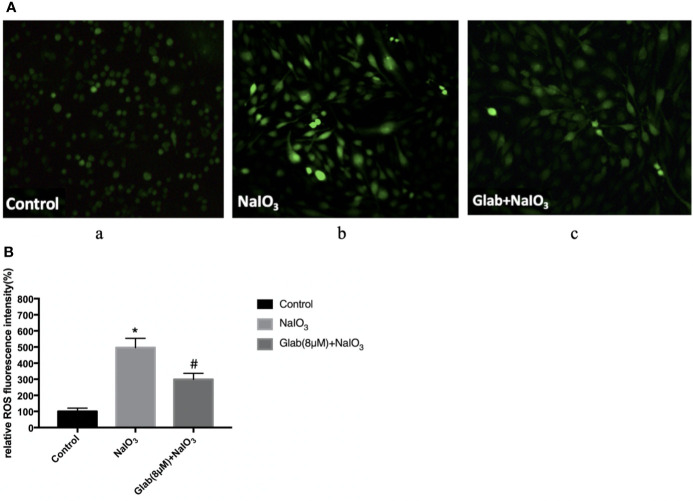
Effects of Glabridin on the production of reactive oxygen species (ROS) in ARPE‐19 cells treated with sodium iodate. **(A)** Dichloro‐dihydro‐fluorescein diacetate (DCFH‐DA) staining of ARPE‐19 cells after 24 h of sodium iodate and Glab treatment. a, retinal pigment epithelium (RPE) treated with DME-F12 (Control); b, RPE treated with sodium iodate(1200μg/ml) (NaIO_3_); c, RPE treated with Glabridin(8μM) plus NaIO_3_ (Glab(8μM)+NaIO_3_). **(B)** ROS levels were significantly increased in the NaIO_3_ group compared with the control group (*P < 0.05), while Glabridin(8μM) significantly decreased ROS levels in ARPE‐19 cells treated with NaIO_3_, compared with NaIO_3_ group (^#^*P < *0.05).

To evaluate the anti-oxidative effect of Glabridin on NaIO_3_-induced ROS production, ARPE-19 cells were treated with NaIO_3_(1200μg/ml) and Glab(8μM) at the same time, incubated for 24 hours and then stained with DCFH-DA. The results showed that ROS production in the NaIO_3_ group was significantly higher than that in the control group. In the treatment group with Glab(8μM), the NaIO_3_ induced ROS production was significantly lower than that in the NaIO_3_ group (**Figure 3**).

### The Protective Effect Of Glabridin on the Retinal Function According to ERG Results

**Figure 4:**
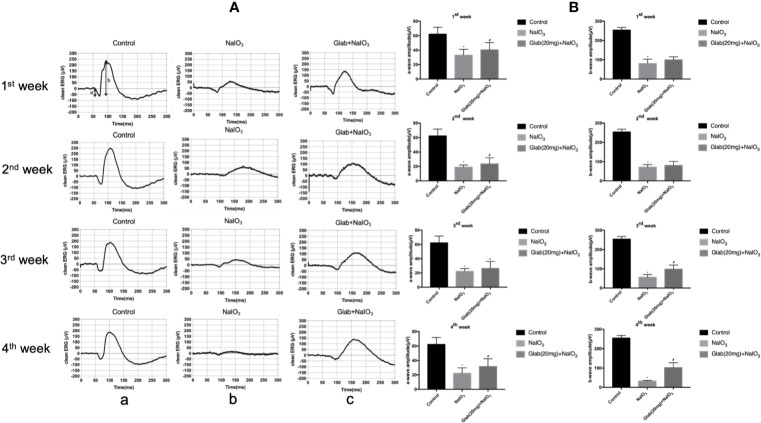
Effects of Glabridin on the retinal function after sodium iodate (NaIO_3_) treatment *in vivo*. **(A)** Electroretinography (ERG) images of changes in the retinal function of mice within four weeks after NaIO_3_ injection and Glab treatment. a, retina of normal mice (Control); b, retina treated with NaIO_3_(25mg/kg) (NaIO_3_); c, retina treated with Glabridin(20mg/kg) plus NaIO_3_ (Glab+NaIO_3_). The degeneration of retinal function in the retina of mice treated with NaIO_3_ was significantly seen throughout 4 weeks. The amplitudes of the waves in the Glab+NaIO_3_ treatment group were significantly higher than those in the NaIO_3_ group throughout 4 weeks suggesting that the retinal function in the retinas of mice treated with Glab was protected from retinal damage induced by NaIO_3_. **(B)** From the first week to the fourth week, the amplitudes of a-wave and b-wave in both the NaIO_3_ group and Glab+NaIO_3_ group were significantly lower than those in the control group (*P < 0.05). The amplitudes of both a-wave and b‐wave in the Glab+ NaIO_3_ group were significantly higher than those in the NaIO_3_ group (^#^*P* < 0.05).

Electroretinogram (ERG) images of the retinas of mice were recorded at 7, 14, 21, and 28 days after NaIO_3_(25mg/kg) injection to both the NaIO_3_ group and Glabridin (Glab) treatment group to monitor the functional changes of the retina over time and compare them among groups. Glabridin (Glab) at a concentration of 20mg/kg was injected intraperitoneally to the NaIO_3_ induced mice daily. ERG recordings of the first week after NaIO_3_ injection showed that the retinal function began to deteriorate due to the damage induced by NaIO_3_ in both groups, while treatment with Glab(20mg/kg) maintained the retinal function compared with the NaIO_3_ group. In the following weeks, the ERG waves in both groups became weaker than those of the first week indicating that NaIO_3_ can damage the retinal function with time. As NaIO_3_ continued to damage the retina in both groups, the retinal function in the NaIO_3_ group was very severely damaged in the third week and the fourth week, whereas treatment with Glab maintained better retinal function than the NaIO_3_ group, indicating that Glab can prevent the retinal degeneration induced by NaIO_3_ (**Figure 4A**). The amplitudes of both a-wave and b-wave in the NaIO_3_ group decreased significantly compared with the control group. The a-wave and b-wave amplitudes in the Glab treatment group were higher than those in the NaIO_3_ group at a significant level, showing that the retinal function was protected by the Glabridin (**Figure 4B**).

### The Protective Effect of Glabridin on the Retina According to OCT Results

**Figure 5:**
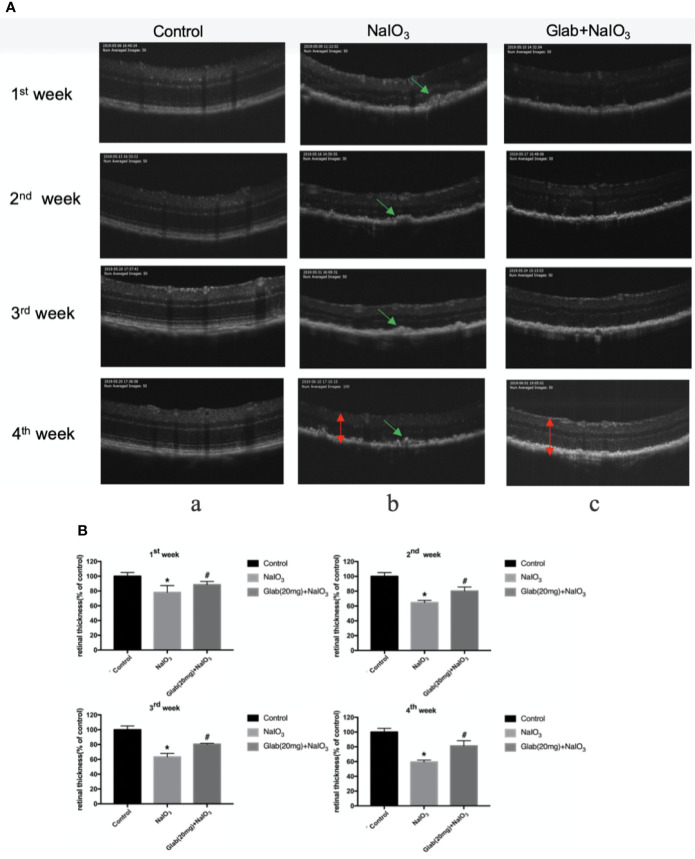
Effects of Glabridin on the thinning of retina and deposits on the retinal pigment epithelial (RPE) layer of the retina monitored with non-invasive imaging *in vivo*. **(A)** Optical coherence tomography(OCT) measurement of the effects of Glabridin on the retinas of mice within four weeks after NaIO_3_ injection and Glab treatment. a, retina of normal mice (Control); b, retina treated with NaIO_3_(25mg/kg) (NaIO_3_); c, retina treated with Glabridin(20mg/kg) plus NaIO_3_ (Glab(20mg)+NaIO_3_). The changes in the thickness and layers of the retina could be seen. The shadows of deposits were significantly seen on the RPE layer in the NaIO_3_ group, while they were not that obvious in the Glab+NaIO_3_ group. **(B)** From the first week to the fourth week, the thinning of the retina was worse week after week in the NaIO_3_ group, compared with the control group (*P < 0.05). Although the degeneration became worse in the NaIO_3_ group, the thickness and layers of the retina in the Glab+NaIO_3_ group remained better than that of the NaIO_3_ group throughout four weeks showing the most significant difference in the third week and fourth week, compared with the NaIO_3_ group (^#^*P < *0.05).

Optical coherence tomography (OCT) images of the retinas of mice were recorded 7, 14, 21, and 28 days after NaIO_3_(25mg/kg) injection to both the NaIO_3_ group and treatment group in order to monitor the structural changes of the retina with non-invasive imaging method. Glabridin (Glab) at a concentration of 20mg/kg was injected intraperitoneally to these NaIO_3_ induced mice daily. In the OCT images of the first week, the damage to the retina was seen as the thickness of the retina of both the NaIO_3_ group and treatment group with Glab(20mg/kg) began thinner than that of the control group and the shadows of bulging deposits in the RPE layer began to appear. Although the differences in NaIO_3_ and Glab treatment groups in the first week and the second week were not very obvious at first, from the third week the damage to the RPE layers and accumulation of deposits in the NaIO_3_-induced model group was significant and the thickness of the retina was significantly reduced, compared with the control group. In contrast, in the treatment group with Glab(20mg/kg) to NaIO_3_-damaged retina, even after the third week and the fourth week, the thickness of the retina was preserved and protected staying almost the same as the first week and the second week. The shadows of deposits were also not significantly seen. These OCT results showed that the NaIO_3_ induced model group continuously inflicted damage to the retina, over time mimicking the retinal degeneration of dry AMD, while the treatment group with Glab significantly protected the retina by reducing the damage induced by NaIO_3_, maintaining the thickness of the retina and reducing bulging deposits (**Figure 5**).

### Histological Results

**Figure 6:**
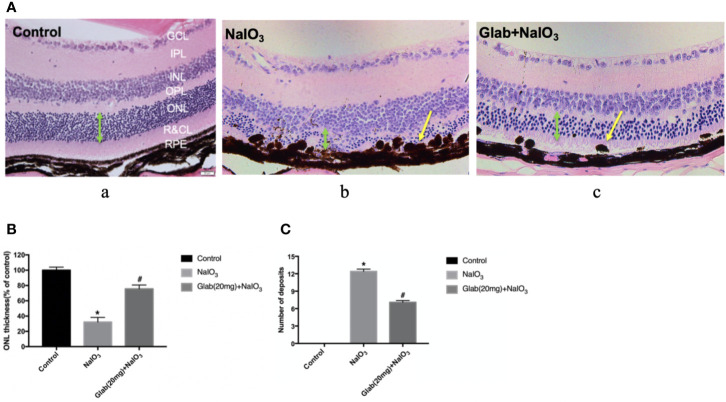
Effects of Glabridin on the retinal layers and number of deposits on the retinal pigment epithelial (RPE) layer as seen in H&E staining *in vivo*. **(A)** Haematoxylin and eosin (H&E) staining of the retina on day 28 after NaIO_3_ and Glab treatment. a, retina of normal mice (Control); b, retina treated with NaIO_3_(25mg/kg) (NaIO_3_); c, retina treated with Glabridin (20mg/kg) plus NaIO_3_ (Glab(20mg)+NaIO_3_). The arrangement of cells in the outer nuclear layer (ONL), rods and cones layer (R&CL), and retinal pigment epithelium(RPE) layer were significantly disordered in the NaIO_3_ group, compared with the control group, while Glab significantly attenuated the disorder of those layers induced by NaIO_3_ in Glab+NaIO_3_ group, compared with NaIO_3_ group. **(B)** The thickness of ONL, the most severely damaged layer, was compared among groups and the results showed that NaIO_3_ significantly reduced the thickness of ONL, compared with the control group (*P < 0.05), while Glab significantly protected the layers and increased the thickness of ONL, compared with NaIO_3_ group (^#^*P < *0.05). **(C)** Both NaIO_3_ and Glab+NaIO_3_ groups significantly increased the number of deposits on the RPE layer, compared with the control group (*P < 0.05), however, Glab significantly decreased the number of deposits on the RPE layer in the Glab+NaIO_3_ group, compared with NaIO_3_ group (^#^*P < *0.05).

After monitoring the retinas of mice with OCT and ERG for 4 weeks, the mice were euthanized and the eyes were harvested to check the histological changes using Haematoxylin and Eosin (H&E) staining. One study about the direct effect of NaIO_3_ on the retina reported that in the histological examination, disorganization of PRs and significant thinning of the entire retina, especially the ONL, were observed along with large regions of complete RPE loss, which had expanded over time (Wang et al., 2014). Our results also showed that the retinal injury induced by NaIO_3_(25mg/kg) after 4 weeks was significant in the retinal pigment epithelium (RPE) layer, rods, and cones layer (R&CL) (also known as photoreceptors(PR) layer), and the outer nuclear layer (ONL), as the arrangement of cells in all these layers was disordered in the NaIO_3_ model group along with the formation of bulging deposits (swelling and bundling of RPE cells) on the retinal pigment epithelium (RPE), in comparison with the control. The arrangement of cells in all those layers was improved in the treatment group with Glab(20mg/ml), and the structure of the RPE layer was significantly protected by reducing the number of deposits compared with the NaIO_3_ model group (**Figure 6**). These histological findings supported the findings of non-invasive imaging of OCT and ERG.

### Expression of Phosphorylation Of ERK1/2 and P38 In Western Blotting Results

**Figure 7:**
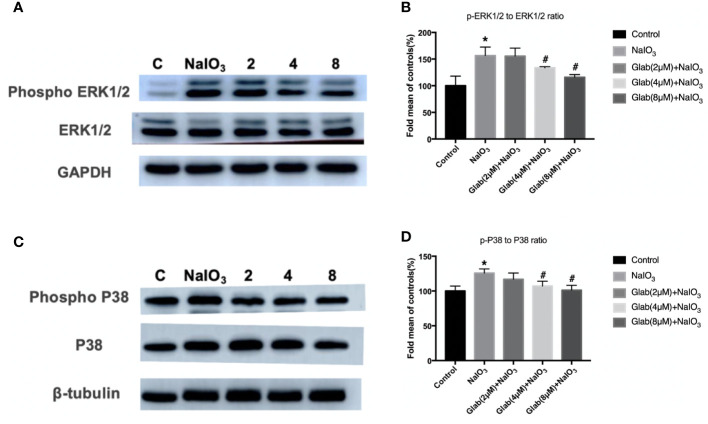
Effects of Glabridin on the protein expression of phosphorylation of ERK1/2 and p38 in ARPE‐19 cells treated with sodium iodate(NaIO_3_) by Western blot detection. Control: retinal pigment epithelial (RPE) cells treated with DME-F12 only, NaIO_3_: RPE cells treated with NaIO_3_(1200 μg/ml), 2,4,8: RPE cells treated with different concentrations of Glab (2,4,8 μM) plus NaIO_3_(Glab+NaIO_3_). **(A)** Effects of Glabridin on the phosphorylation of ERK1/2. **(B)** The expression of phosphorylated ERK1/2 was significantly increased in the NaIO_3_ group, compared with the control group(*P < 0.05), while Glabridin significantly decreased the phosphorylation of ERK1/2 at the concentration of 4 and 8 μmol, compared with NaIO_3_ group (^#^*P* < 0.05). **(C)** Effects of Glabridin on the phosphorylation of p38. **(D)** The expression of phosphorylated p38 was significantly increased in the NaIO_3_ group, compared with the control group (*P < 0.05), while Glabridin significantly decreased the phosphorylation of p38 at the concentration of 4 and 8 μmol, compared with NaIO_3_ group (^#^*P* < 0.05).

ERK1/2 and P38 MAPK pathway regulates cell proliferation, cell differentiation, and cell death and is reported to be closely related to oxidative stress, inflammation, and aging. The Western blotting results showed that the phosphorylation of ERK1/2 and P38 were significantly increased in the NaIO_3_ group compared with the control. In the treatment group of Glab(4,8 μM), the phosphorylation of ERK1/2 and P38 was significantly decreased compared with the NaIO_3_ group (**Figure 7**).

## Discussion

Since dry AMD is untreatable, the best current options are to slow down or stop the progress of the disease. Following the present understanding of AMD mechanisms, retinal pigment epithelial (RPE) cell injury plays a very important role in the pathogenesis of AMD. This study investigated a preventive method for AMD at the cellular level, by considering the effects of Glabridin (Glab) in ARPE-19 cells damaged by sodium iodate (NaIO_3_). Glab prevented the excessive overproduction of reactive oxygen species (ROS), suggesting that Glab can prevent oxidative stress in RPE cells. Hoechst33342 results showed that Glab could prevent the apoptosis of ARPE-19 cells and flow cytometry results supported that Glab can reduce apoptotic cells induced by NaIO_3_.

To detect and monitor the damage on the retina and how much Glab can prevent the retinal injury caused by NaIO_3_, optical coherence tomography(OCT), and electroretinography(ERG) of the retinas of the mice were recorded every week up until 28 days. OCT results showed that in the first week of NaIO_3_ injection, the layers of retina began to be disordered in both the NaIO_3_ and the treatment group and the shadows of the deposits on the RPE layer started to appear in the NaIO_3_ group. In the second week, the retinal layers in the treatment group were also damaged by NaIO_3_ but were not as bad as those of the NaIO_3_ group. In the third and fourth weeks, the protective effect of Glab could be seen significantly in the treatment group compared with the NaIO_3_ group. The retinal thickness became so thin in the NaIO_3_ group while treatment with Glab maintained the thickness of the retina to almost the same extent as in the first and second week. The shadows of deposits in the treatment group were also fewer than those in the NaIO_3_ group.

In ERG images, a-wave and b-wave amplitudes in the treatment group were higher than those in the NaIO_3_ group throughout 4 weeks. The a-wave, initial corneal-negative deflection, derived from the cones and rods of the outer photoreceptor layers, is a measure of the photoreceptor response, and the b-wave, corneal-positive deflection, is derived from the inner retina, predominantly Muller and ON-bipolar cells. The b-wave is the most common component of the ERG used in the clinical and experimental analysis of human retinal function (Asi & Perlman, 1992; Berrow et al., 2010). There were obvious changes in both the a-wave and b-wave amplitudes, which became lower with time, demonstrating that NaIO_3_ damaged the retinal function gradually and caused degeneration very severely in the fourth week. In contrast, the amplitudes of the a-wave and b-wave in the treatment group with Glab were significantly higher and maintained at almost the same level as the first week, even 4 weeks after the NaIO_3_ injection. The results of the non-invasive investigation with both OCT and ERG supported the fact that Glab could maintain the morphology as well as the function of the retina. In H&E staining, the photoreceptor cells (PR) layer and outer nuclear layer (ONL) were severely disrupted and a large number of deposits were seen on the RPE layer in the NaIO_3_ group. In the treatment group with Glab, only a little disruption in the PR layer and a significantly lower number of deposits on the RPE layer were seen, compared with the NaIO_3_ group. The histological findings supported the results of the non-invasive OCT and ERG. These results indicate that NaIO_3_(25mg/kg) severely damaged the retina of the C57BL/6J mice mainly in the ONL, PR, and RPE layers after 4 weeks, and that Glabridin (Ip, 20mg/kg.d) could relieve the progression of this damage and prevent retinal degeneration over time.

The mitogen-activated protein kinase (MAPK) pathway transduces signals to the cell nucleus to regulate transcription and influences cell proliferation, differentiation, and apoptosis through activation of the protein kinase cascades (Morrison, 2012; Jiang and Tang, 2018). ERK1/2 (extracellular signal-regulated protein kinase) is a member of the MAPK family and has an important role in delivering extracellular signals to the nucleus, and thus regulating the cell cycle, cell proliferation, and cell development. There is evidence that ERK1/2 is closely associated with cell aging (Zou et al., 2019). The ERK1/2 pathway is assumed to be a direct regulator of the visual cycle and a critical component of the viability of RPE and photoreceptor cells (Pyakurel et al., 2017). One study has suggested that ERK1/2 activation triggers RPE cell death in dry AMD, acting in accordance with the chronic nature of AMD progression (Dridi et al., 2012). Another main member of the MAPK pathway, p38 is more responsive to stress stimuli ranging from osmotic shock and ionizing radiation to cytokine stimulation. In mammalian cells, the p38 isoforms are strongly activated by environmental stresses and inflammatory cytokines but not appreciably by mitogenic stimuli. Many other studies have indicated that p38 activity is critical for oxidative stress, aging, and inflammatory response (Lee et al., 1994; Roux and Blenis, 2004). In the present study, the RPE cell death induced by NaIO_3_ increased the phosphorylation levels of ERK1/2 and p38 whereas the phosphorylation levels of ERK1/2 and p38 were decreased when treated with Glab. This finding indicated that Glabridin, a flavonoid extracted from licorice root, could prevent RPE cell death by inhibiting the phosphorylation of ERK1/2 and the p38 MAPK pathway.

Although we found that Glab protected against NaIO_3_ induced-RPE and retina injury, some of the limitations of this study need to be addressed. The method of extended intraperitoneal administration of Glab was used in this study, however, the pharmacokinetic parameters of Glab in the retina were not obtained. It is necessary to study its delivery/penetration into the retina by administering the drug intravitreally. The high and extended dose of NaIO_3_ used in this study would likely cause inflammation in the RPE cells and retina, the inflammatory cytokines should be measured to define the anti-inflammatory effects of Glab. Furthermore, the mechanisms of how Glab works on retinal degeneration are still unknown and needed to be studied in areas other than ERK1/2 and the p38 MAPK pathway. The mitochondrial ROS (MitoSOX) activation of Caspase 3 and other possible signaling pathways should also be studied in further experiments. Therefore, the findings in this study are preliminary, and further work is needed to address the role of inflammation, pharmacokinetics after ocular administration, and there need to be more stringent studies on other signaling pathways in the future, to gain a better understanding of Glab’s utility in dry AMD.

As seen in this study, while NaIO_3_ worsens the retinal damage week by week, Glabridin could prevent retinal degeneration and maintain it in the same condition as the first week of damage induced by NaIO_3_. This indicates that while retinal degeneration progresses over time, Glabridin could delay or even stop the progress. According to available evidence, the main features of dry AMD are mainly associated with oxidative stress and the apoptosis of RPE cells caused by aging. ERK1/2 and the p38 MAPK pathway are closely related to those features. This study proved that Glabridin could prevent the retinal degeneration and RPE cell death caused by NaIO_3_ with the mechanism of inhibiting the phosphorylation of ERK1/2 and p38 MAPK, and suggested that further experiments should be done on this drug, examining its potential use and clinical applications for the prevention and treatment of dry AMD.

## Data Availability Statement

The raw data supporting the conclusions of this article will be made available by the authors, without undue reservation.

## Ethics Statement

The animal study was reviewed and approved by Experimental Animal Ethics Committee of Jinzhou Medical University.

## Author Contributions

KA: designed and performed most of the experiments and contributed to the manuscript. KA: undertook methodology, investigation, validation, Visualization, formal analysis, and writing of the original draft. ZK: contributed to the methodology, investigation, and curation of data. SJ: was involved with conceptualization, and formal analysis. JH: was involved with the supervision, conceptualization, project administration, writing, review, and editing of the manuscript. HL: worked on supervision, resources, project administration, and funding acquisition. All authors contributed to the article and approved the submitted version.

## Funding

This work was supported in part by grants from the Natural Science Foundation of Liaoning Province (201602287) and Liaoning Distinguished Professor Project to JH (XLYC1802113) and HL (XLYC1802039).

## Conflict of Interest

The authors declare that the research was conducted in the absence of any commercial or financial relationships that could be construed as a potential conflict of interest.

## Abbreviations

Age-related macular degeneration (AMD), Electroretinography (ERG), Glabridin (Glab), Haematoxylin and eosin (H&E), Mitogen-activated protein kinase (MAPK), 3-(4,5-dimethylthiazol-2-yl)-2,5-diphenyl tetrazolium bromide (MTT), Optical Coherence Tomography (OCT), Outer Nuclear Layer (ONL), Photoreceptor cells (PR), Reactive Oxygen Species (ROS), Retinal Pigment Epithelial (RPE) cells, Sodium Iodate (NaIO_3_).

## References

[B1] Ambati J. Fowler B. J. (2012). Mechanisms of age-related macular degeneration. Neuron 75 (1), 26–39. 10.1016/j.neuron.2012.06.018 22794258 PMC3404137

[B2] Asi H. Perlman I. (1992). Relationships between the electroretinogram a-wave, b-wave and oscillatory potentials and their application to clinical diagnosis. Doc. Ophthalmol. 79 (2), 125–139. 10.1007/BF00156572 1591967

[B3] Berrow E. J. Bartlett H. E. Eperjesi F. Gibson J. M. (2010). The electroretinogram: A useful tool for evaluating age-related macular disease? Doc. Ophthalmol. 121 (1), 51–62. 10.1007/s10633-010-9226-1 20232109

[B4] Bowes Rickman C. Farsiu S. Toth C. A. Klingeborn M. (2013). Dry age-related macular degeneration: Mechanisms, therapeutic targets, and imaging. Investig. Ophthalmol. Vis. Sci. 54 (14), ORSF68–ORSF80. 10.1167/iovs.13-12757 PMC3864379 24335072

[B5] Bungau S. Abdel-Daim M. M. Tit D. M. Ghanem E. Sato S. Maruyama-Inoue M. (2019). Health Benefits of Polyphenols and Carotenoids in Age-Related Eye Diseases. Oxid. Med. Cell. Longevity 2019, 22. 10.1155/2019/9783429 PMC6390265 30891116

[B6] Carmeli E. Fogelman Y. (2009). Antioxidant effect of polyphenolic glabridin on LDL oxidation. Toxicol. Ind. Health 25 (4-5), 321–324. 10.1177/0748233709103034 19651803

[B7] Colijn J. M. Buitendijk G. H. S. Prokofyeva E. Alves D. Cachulo M. L. Khawaja A. P. (2017). Prevalence of Age-Related Macular Degeneration in Europe: The Past and the Future. Ophthalmology 124 (12), 1753–1763. 10.1016/j.ophtha.2017.05.035 28712657 PMC5755466

[B8] Dridi S. Hirano Y. Tarallo V. Kim Y. Fowler B. J. Ambati B. K. (2012). ERK1/2 activation is a therapeutic target in age-related macular degeneration. Proc. Natl. Acad. Sci. U. S. A. 109 (34), 13781–13786. 10.1073/pnas.1206494109 22869729 PMC3427082

[B9] Enzmann V. Row B. W. Yamauchi Y. Kheirandish L. Gozal D. Kaplan H. J. (2006). Behavioral and anatomical abnormalities in a sodium iodate-induced model of retinal pigment epithelium degeneration. Exp. Eye Res. 82 (3), 441–448. 10.1016/j.exer.2005.08.002 16171805

[B10] Handa J. T. Cano M. Wang L. Datta S. Liu T. (2017). Lipids, oxidized lipids, oxidation-specific epitopes, and Age-related Macular Degeneration. Biochim. Biophys. Acta Mol. Cell Biol. Lipids. 1862 (4), 430–440. 10.1016/j.bbalip.2016.07.013 27480216 PMC5280582

[B11] Hanneken A. Lin F. F. Johnson J. Maher P. (2006). Flavonoids protect human retinal pigment epithelial cells from oxidative-stress-induced death. Invest. Ophthalmol. Visual Sci. 47, 3164–3177. 10.1167/iovs.04-1369 16799064

[B12] He H. Wei D. Liu H. Zhu C. Lu Y. Ke Z. (2019). Glycyrrhizin protects against sodium iodate-induced RPE and retinal injury though activation of AKT and Nrf2/HO-1 pathway. J. Cell. Mol. Med. 23 (5), 3495–3504. 10.1111/jcmm.14246 30821111 PMC6484410

[B13] Huang H. L. Hsieh M. J. Chien M. H. Chen H. Y. Yang S. F. Hsiao P. C. (2014). Glabridin mediate caspases activation and induces apoptosis through JNK1/2 and p38 MAPK pathway in human promyelocytic leukemia cells. PloS One 9 (6), e98943. 10.1371/journal.pone.0098943 24901249 PMC4047044

[B14] Huang K. Liu Y. Tang H. Qiu M. Li C. Duan C. (2019). Glabridin prevents doxorubicin-induced cardiotoxicity through gut microbiota modulation and colonic macrophage polarization in mice. Front. Pharmacol. 10, 107. 10.3389/fphar.2019.00107 30833897 PMC6387923

[B15] Jiang L. Tang Z. (2018). Expression and regulation of the ERK1/2 and p38 MAPK signaling pathways in periodontal tissue remodeling of orthodontic tooth movement. Mol. Med. Rep. 17 (1), 1499–1506. 10.3892/mmr.2017.8021 29138812 PMC5780090

[B16] Lee J. C. Laydon J. T. McDonnell P. C. Gallagher T. F. Kumar S. Green D. (1994). A protein kinase involved in the regulation of inflammatory cytokine biosynthesis. Nature 372 (6508), 739–746. 10.1038/372739a0 7997261

[B17] Li S. Gaur U. Chong C. M. Lin S. Fang J. Zeng Z. (2018). Berberine protects human retinal pigment epithelial cells from hydrogen peroxide-induced oxidative damage through activation of AMPK. Int. J. Mol. Sci. 19 (6), pii: E1736. 10.3390/ijms19061736 PMC6032421 29895743

[B18] Lim L. S. Mitchell P. Seddon J. M. Holz F. G. Wong T. Y. (2012). Age-related macular degeneration. Lancet 392 (10153), 1147–1159. 10.1016/S0140-6736(12)60282-7 30303083

[B19] Maher P. Hanneken A. (2005). Flavonoids protect retinal ganglion cells from oxidative stress-induced death. Invest. Ophthalmol. Visual Sci. 46, 4796–4803. 10.1167/iovs.05-0397 16303981

[B20] Mizota A. Adachi-Usami E. (1997). Functional recovery of retina after sodium iodate injection in mice. Vision Res. 37 (14), 1859–1865. 10.1016/S0042-6989(97)00015-1 9274771

[B21] Morrison D. K. (2012). MAP kinase pathways. Cold Spring Harb. Perspect. Biol. 4 (11), pii: a011254. 10.1101/cshperspect.a011254 PMC3536342 23125017

[B22] Nowak J. Z. (2006). Age-related macular degeneration (AMD): Pathogenesis and therapy. Harmacol. Rep. 58 (3), 353–363. 16845209

[B23] Pyakurel A. Balmer D. Saba-El-Leil M. K. Kizilyaprak C. Daraspe J. Humbel B. M. (2017). Loss of Extracellular Signal-Regulated Kinase 1/2 in the Retinal Pigment Epithelium Leads to RPE65 Decrease and Retinal Degeneration. Mol. Cell. Biol. 37 (24), pii: e00295–17. 10.1128/mcb.00295-17 PMC5705814 29038159

[B24] Roux P. P. Blenis J. (2004). ERK and p38 MAPK-Activated Protein Kinases: a Family of Protein Kinases with Diverse Biological Functions. Microbiol. Mol. Biol. Rev. 68 (2), 320–344. 10.1128/mmbr.68.2.320-344.2004 15187187 PMC419926

[B25] Silván J. M. Reguero M. De Pascual-Teresa S. (2016). A protective effect of anthocyanins and xanthophylls on UVB-induced damage in retinal pigment epithelial cells. Food Funct. 7 (2), 1067–1076. 10.1039/c5fo01368b 26781209

[B26] Wang J. Iacovelli J. Spencer C. Saint-Geniez M. (2014). Direct effect of sodium iodate on neurosensory retina. Investig. Ophthalmol. Vis. Sci. 55 (3), 1941–1953. 10.1167/iovs.13-13075 24481259 PMC4049579

[B27] Wang G. Sun G. Wang Y. Yu P. Wang X. Zhou B. (2018). Glabridin attenuates endothelial dysfunction and permeability, possibly via the MLCK/p−MLC signaling pathway. Exp. Ther. Med. 17 (1), 107–114. 10.3892/etm.2018.6903 30651770 PMC6307408

[B28] Zeiss C. J. (2013). Animal Models of Age-Related Macular Degeneration. Anim. Models Study Hum. Dis. 10.1016/B978-0-12-415894-8.00005-1

[B29] Zhang X. Y. Ng T. K. Brelén M. E. Wu D. Wang J. X. Chan K. P. (2016). Continuous exposure to non-lethal doses of sodium iodate induces retinal pigment epithelial cell dysfunction. Sci. Rep. 6, 37279. 10.1038/srep37279 27849035 PMC5110957

[B30] Zhang L. P. Zhao Y. Liu G. J. Yang D. G. Dong Y. H. Zhou L. H. (2017). Glabridin attenuates lipopolysaccharide-induced acute lung injury by inhibiting p38MAPK/ERK signaling pathway. Oncotarget 8 (12), 18935–18942. 10.18632/oncotarget.14277 28039487 PMC5386659

[B31] Zhang L. Chen H. Wang M. Song X. Ding F. Zhu J. (2018). Effects of glabridin combined with 5-fluorouracil on the proliferation and apoptosis of gastric cancer cells. Oncol. Lett. 15 (5), 7037–7045. 10.3892/ol.2018.8260 29725429 PMC5920351

[B32] Zou J. Lei T. Guo P. Yu J. Xu Q. Luo Y. (2019). Mechanisms shaping the role of ERK1/2 in cellular senescence (Review). Mol. Med. Rep. 19 (2), 759–770. 10.3892/mmr.2018.9712 30535440 PMC6323238

