# Supplementary figures and images for: Glabridin Attenuates the Retinal Degeneration Induced by Sodium Iodate In Vitro and In Vivo
Source: Front Pharmacol. 2020 Oct 15;11:566699. doi: 10.3389/fphar.2020.566699 (PMC7593553; doi:10.3389/fphar.2020.566699)

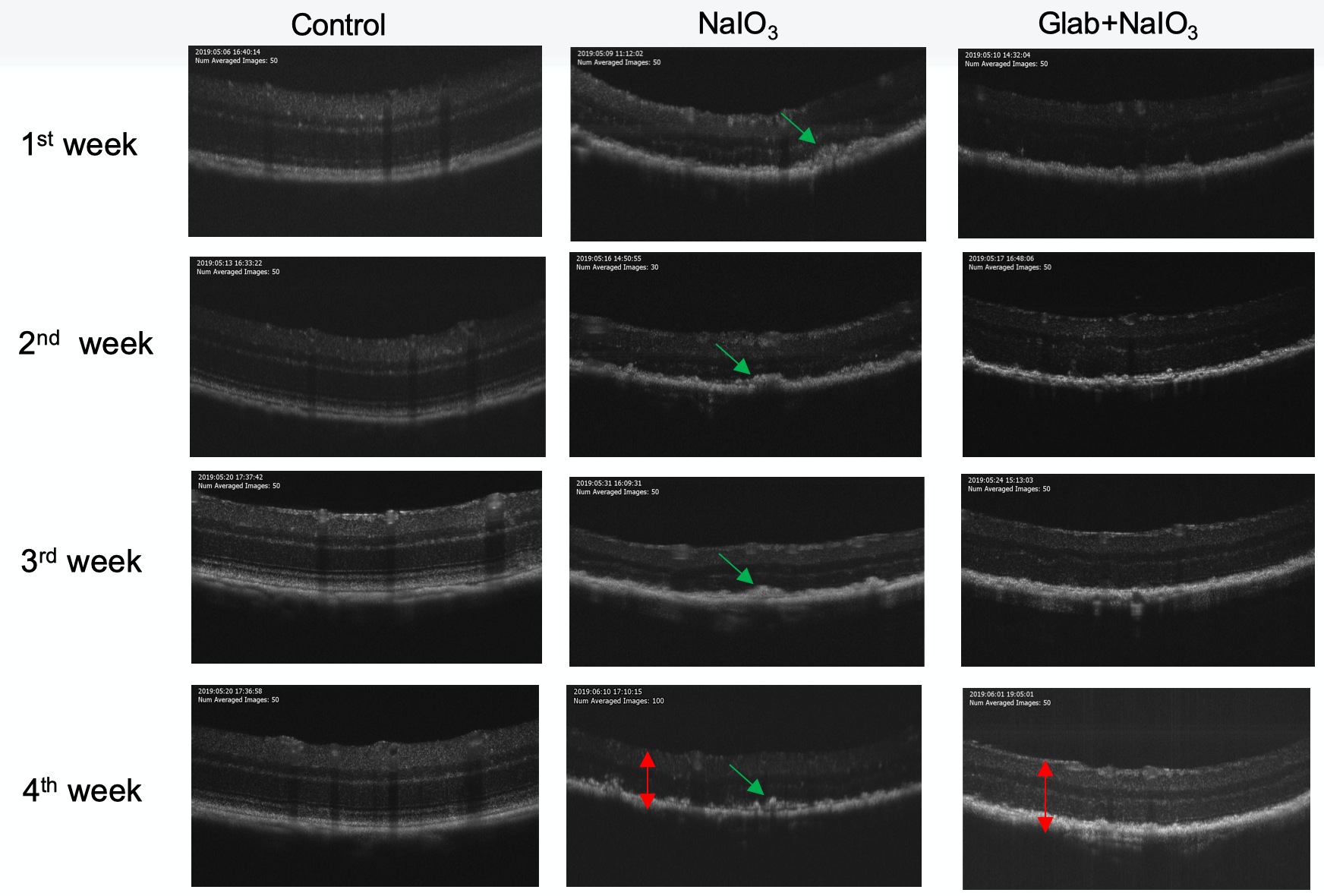

Supplement: Supplementary file 1 [file Image_1.png]
